# Supplementary material for: Evoked temporal summation in dogs to assess pain central sensitization and modulation – A feasibility study
Source: PLoS One. 2026 Jun 2;21(6):e0349863. doi: 10.1371/journal.pone.0349863 (PMC13229318; doi:10.1371/journal.pone.0349863)
Supplement: Attachment — Submitted filename: Response to Reviewers 16-04-26.docx [file pone.0349863.s002.docx]

**Review – Comments to the Author**

**Reviewer #1**

I read the manuscript submitted by the authors with great interest and attention. It is an original work that provides relevant data in the field. However, I have raised certain points that require clarification.

**Author response**: Thank you for your precious feedback and interest in our work. We have carefully addressed all of your comments. Thank you for your valuable suggestions, we believe they have improved the clarity of the manuscript. We hope the revised version meets your expectations.

**Abstract section**

Comment 1: Lines 31 and 32: the use of the abbreviation Newton (N) is confusing. I had to wait until the methods section to make the connection. It would be clearer to either specify or write out the term in full.

**Author response**: Thank you for pointing this out. The full term was defined in the abstract (Line 31) and also specified in Table 1 (Line 152) and in Material and Methods (Line 158).

Comment 2: It is unclear why the authors use a threshold of 7% for inhibition functionality. In the methods section, it is reported as ‘according to internal data,’ but I think this should be explained further.

**Author response**: Thank you for this comment. The threshold was established based on internal data that are the object of another manuscript currently submitted in another journal. We agree this was confusing, as such presented. To improve clarity and transparency, we have added details as follows: “A negative value with TSP meant facilitation, whereas a functional inhibition with CPM was determined when the percentage of change was positive and greater than 7% (internal data 2021, healthy dogs median ratio of 7%) or a maximum threshold (10N) was reached” (Lines 196–198).

**Introduction section**

Comment 3: line 61. This manuscript deals with temporal summation; I will not address spatial summation, which is a separate process and has not been tested in the manuscript.

**Author response**: Thank you for your comment. To avoid any misunderstanding, the term “spatial” was removed (Line 61).

Comment 4: Line 70: I am torn about the comments you mentioned. I agree with you that past studies using nociceptive reflexes are not QST. That being said, I do not think that in your study you are measuring the conscious perception of pain and its affective-emotional component, since you are observing behaviour. I would tend to reiterate this point.

**Author response**: Thank you for your comment. Indeed, QST does not directly measure the motivational-affective component of pain; however, pain perception must be inferred from behavioral responses. In the present study to minimize bias, stimulation was stopped only after the observation of two consistent behavioral responses, and a reliability score was applied to ensure that reactions were not attributable to environmental stimuli. Further, the behavioral endpoints reported (*e.g.* withdrawal, vocalization) are not simple reflexes but involve voluntary, motivated responses. As such, these aversive behaviors integrate nociceptive input with higher‑order processing, including motivational-affective states such as discomfort, stress, or anxiety. Clarifications were added in the corresponding sections (Lines 71 and 169).

**Method section**

Comment 5: (just out of curiosity) I understand the importance of selecting dogs that obey and respond to commands such as sit or lie down, but with OA, doesn't that immediately exclude overly sensitive dogs that would have been interesting to include in order to have dogs with extreme responses?

**Author response**: Thank you for your comment. For this feasibility study, the inclusion criteria focused on ensuring that dogs were able to remain in a stable and standardized position to allow reliable implementation of TSP protocol. Osteoarthritis itself did interfere minimally with the ability to sit or lay down (in our dog colony). The (over)sensitivity of the OA dogs was not a discriminatory factor. We can assure the Reviewer that several OA dogs were overly sensitive at the inclusion in the cohort, and they required lot of tender care and attention to improve their sociability. Rather, prior training, the dog’s confidence in humans, and its level of anxiety were the main factors influencing how easily a dog could remain still during the evaluation.

Comment 6: The protocol has many elements, and I think a diagram that visually represents the two parts of the protocol with the stimulation parameters would be helpful in better understanding and following the sequence of elements.

**Author response**: Thank you for your suggestion. An additional Figure was included (Fig 2. Timeline of the experimental design) to support Table 1 protocol descriptions. (Line 180).

Comment 7: Table 1 - there are more dogs in the tail stimulation protocol than in the ‘forearm’ protocol, so I wonder if the same healthy dogs were used in both protocols. This is not clear in the text.

**Author response**: Thank you for your comment. The same cohort of healthy dogs was included for all stimulation protocols, except for one dog. As shown in Table 2, there was a slight difference in age of the dogs between both protocols. This point was clarified in the revised manuscript as follows: “The same healthy dogs were included in all stimulation protocols, except for one dog during the tail-base stimulations (Protocols E and F).” (Line 150). And you are right, we were able to include more OA dogs for the tail base than the forearm stimulations, as more dogs completed their acclimation.

Comment 8: If I understand correctly, the clinical outcome of TSP is the dog's aversive reaction. I think the different reactions should be presented more precisely (in a table, for example) and reported in the results section, as there may be an association between the dog's sensitivity and its reaction.

**Author response**: Thank you for this insightful comment. You are correct in pointing out that individual behavioral responses could reflect differences in TSP sensitivity and, generally each dog response remained consistent throughout the protocol. However, badly reactions were not reported individually for each evaluation; we focused on the number of stimulations required to elicit the aversive responses. For sure, a more detailed description of the reactions would be highly relevant in future studies.

Comment 8 bis: In this regard, I was wondering if the authors had considered taking physiological measurements such as heart rate or breathing rate?

**Author response**: Thank you for this relevant comment. Although physiological parameters such as heart rate or breathing rate were not assessed in this study, they can represent promising objective outcomes to predict aversive reactions during TSP. Establishing correlations between some physiological parameters and central sensitization could contribute to the development of a clinical assessment of neuro-sensitization. Recent studies in young people with fibromyalgia have demonstrated reduced heart rate variability associated with increased pain levels (Diomeda *et al*., 2025). However, a lack of specificity was also reported, as heart rate variability was also correlated with anxiety. Further studies are necessary to determine the significance of these parameters in understanding central sensitization in dogs.

Diomeda, F., Dell'Anna, B., Greco, R., et al. (2025). Heart rate variability (HRV) in juvenile fibromyalgia: a preliminary study. Pediatr Rheumatol Online J *23*, 114. doi : 10.1186/s12969-025-01163-7.

Comment 9: Line 194: more information is needed on the 7% threshold.

**Author response**: Thank you for the comment. The threshold was established based on internal data that were collected in a longitudinal study, object of another manuscript currently submitted. To improve clarity and transparency, we added the following details: “A negative value with TSP meant facilitation, whereas a functional inhibition with CPM was determined when the percentage of change was positive and greater than 7% (internal data 2021, healthy dogs median ratio of 7%) or a maximum threshold (10N) was reached” (Line 194–197).

Comment 10: Perhaps I missed it, but was it the same person who performed all the tests for the ICCs?

**Author response**: Thank you for pointing this out. Yes, the same evaluators (A.DE. and M.B.-G.) performed all the tests.

**Results section**

Comment 11: There are very few healthy dogs compared to OA dogs. Is there a reason for such a small sample size?

**Author response**: This is a judicious comment. The limited number of healthy dogs was related to the ethical considerations for housing healthy individuals within a research colony. Additionally, at the start of the colony, more healthy dogs were included, but part of them developed OA over the years and were therefore displaced to the OA chronic pain group, reducing the healthy group to *N* = 4.

Comment 12: Line 276: explain what RMTS is.

**Author response:** Thank you for pointing this out. The term was removed as it corresponds to Response to Mechanical Temporal Summation (RMTS) (not used in the present manuscript) and was modified by “mechanical stimulations” (Line 277).

**Discussion section**

Comment 13: Line 416 concerning the fact that dogs are neutered. I may add a section on the role of reproductive hormones in pain perception/modulation.

**Author response**: Thank you for this suggestion. Some details were added in the Discussion section, but without going into too much depth, since that was not the focus of this study. The paragraph was modified as follows: “All individuals have been neutered, but not at the same age, which could give rise to acquired behaviors. Indeed, sexual hormone such as estrogen has demonstrated a protective role against the development of central sensitization [52], whereas early neutering (< 6 months) can accelerate the development of OA in dogs [53] as well as in humans [54]. Even though this is a potential bias, this reflects the real challenge in clinics.” (Lines 419–422)

Comment 14: Given that the aim of the project is to determine the feasibility of a TSP protocol in dogs and that several parameters/populations have been tested, I will add a section along the lines of ‘based on our results, here is what we would recommend for measuring TSP in dogs’.

**Author response**: Thank you for the relevant suggestion. An additional sentence was included in the Discussion section as follows: “Overall, repeated mechanical stimuli under nociceptive threshold at tail-base for 0.50Hz and 2N is recommended for measuring TSP and induced facilitation in dogs. However, the response to mechanical TSP looks like a complementary assessment method, […] but does not correlate linearly with functional impairment.” (Lines 409–413). Further, to improve clarity of the Discussion section each Protocol was included in its corresponding interpretation (Lines 333, 334, 347, 348, 355, 357 and 358).

Comment 15: I will also mention the potential clinical implications of this protocol. This aspect is glossed over too quickly in the conclusion.

**Author response**: Thank you for your comment. Some clarifications were added as follows: “This study represents one of the first steps toward characterizing canine OA pain phenotypes according to neuro sensitization. Although the small number of healthy dogs was a limitation, the group homogeneity made it possible to distinguish TSP specificity compared to OA dogs. The next steps will be to confirm specificity, validate the inter-rater’s reproducibility and determine the sensitivity of response to mechanical TSP and CPM to different treatments in dogs. Although the direct clinical application of QST to assess neuro sensitization is currently limited by factors such as stress induced analgesia in clinical settings, future research should aim to develop and validate alternative assessments that are easier to implement in routine practice. Identifying F/I balance will contribute to personalized treatments, by reinforcing inhibitory control, a predictive marker of responsiveness to non-steroidal anti-inflammatory drug in humans [57].” (Lines 434–443)

**Reviewer #2**

I thank the authors for submitting their manuscript. The research addresses an innovative and significant topic for both the journal and clinical practice, promising a substantial impact on enriching current knowledge. This work presents significant advances in the field and highlights a topic of interest to readers. The proposed methodology includes essential elements of the research; however, certain aspects of the manuscript need clarification. I have included some comments that I hope will be useful to the authors.

**Author response**: Thank you for your precious feedback and for highlighting the innovative aspect of our work. We have made some clarifications and hope the revised version to meet your expectations.

**General comments**

Comment 1: L128: Was the quality of life evaluated using a validated scale? If not, please mention this as a limitation.

**Author response**: Thank you for your comment. The quality of life was not directly assessed in this study. Pain scales such as the canine Brief Pain Inventory (CBPI) and DoloDog were used in another project (two-year longitudinal study), and those results were reported in a separate manuscript. However, the inclusion of a dedicated quality of life scale would be of interest to determine the potential influence of pain profile. A sentence was added in the limitation part of the Discussion: “Chronic OA pain was previously associated with a decreased quality of life [57], it could be of interest to assess the influence of neuro-sensitization and F/I balance on dogs quality of life in future studies.” (Lines 431–433)

Comment 2: L199: Please include the analysis of statistical normality and the procedure used for determining the sample size.

**Author response**: Thank you for your suggestion. Given the small sample size (particularly for healthy dogs), assumptions of normality could not be reliably verified. Further, TSP protocol generated ordinal data (scores ranging from 0 to 30). Therefore, non-parametric statistical tests were used (Mann-Whitney *U* test, Spearman rank test correlation and Fisher’s exact test) as described in the Statistical Approaches section (Lines 201–202, 211).

The sample size was determined by the number of dogs with a compliant behavior and ability to respond to commands. It corresponded to more than half of the osteoarthritic dogs and all available healthy dogs in the research colony. This study was designed as a methodological exploration to assess TSP tolerability and to identify potential differences between healthy and osteoarthritic dogs. We acknowledge that the small number of healthy dogs is a limitation, and a sentence was added as follows in the Discussion section: “Although the small number of healthy dogs was a limitation, the group homogeneity made it possible to distinguish TSP specificity compared to OA dogs.” (Lines 435–436)
